# The Role of Symbiotic Nitrogen Fixation in Sustainable Production of Biofuels

**DOI:** 10.3390/ijms15057380

**Published:** 2014-04-29

**Authors:** Bandana Biswas, Peter M. Gresshoff

**Affiliations:** Centre for Integrative Legume Research (CILR), the University of Queensland, St Lucia Brisbane, QLD 4072, Australia; E-Mail: p.gresshoff@uq.edu.au

**Keywords:** biofuel, legumes, life-cycle analysis, nitrogen fixation

## Abstract

With the ever-increasing population of the world (expected to reach 9.6 billion by 2050), and altered life style, comes an increased demand for food, fuel and fiber. However, scarcity of land, water and energy accompanied by climate change means that to produce enough to meet the demands is getting increasingly challenging. Today we must use every avenue from science and technology available to address these challenges. The natural process of symbiotic nitrogen fixation, whereby plants such as legumes fix atmospheric nitrogen gas to ammonia, usable by plants can have a substantial impact as it is found in nature, has low environmental and economic costs and is broadly established. Here we look at the importance of symbiotic nitrogen fixation in the production of biofuel feedstocks; how this process can address major challenges, how improving nitrogen fixation is essential, and what we can do about it.

## 1. Introduction

Two of humanity’s major needs are food and energy. With the human population rising at an alarming rate (1 billion increase from present 7.2 billion in the next 12 years (UN estimate)), food security, mainly due to increasing scarcity of land and water resources has become a major political and scientific concern. Ever since humans transitioned from hunter-gatherers to a stable agriculture-based society, improvement of crops has been a major goal. Early farmers observed that land became less productive when planted year after year and concluded that plants absorbed certain nutrients from the soil. In the 1730s, crop rotation was implemented in Europe as a method to improve productivity of major crops. However, it was not until the mid-1800s that the understanding of plant nutrition had advanced enough to realise the importance of added nitrogen and phosphorus to the soil. The German chemist Justus von Liebig was the first to promote the importance of ammonia and inorganic minerals to plant nutrition and developed the first commercial fertiliser by treating phosphate of lime in bone meal with sulphuric acid. Although this failed, because of not being properly absorbed by the crops, it started a trend in fertiliser development. The early fertilisers were mainly based on manures and the effects of many types of manure on plant growth were tested and used. The Rothamsted (UK) Research Station, started at this time by the British entrepreneur John Bennet Lawes and Joseph Gilbert (a student of von Liebig) is still involved in the study of the effect of organic and inorganic fertilisers on crop yield.

However, it was not until the process of atmospheric nitrogen fixation was established, first by Henry Cavendish in 1784, that synthetic fertilisers became widespread. His process was soon replaced by a more efficient Haber-Bosch process, which revolutionised agriculture and won the inventors Fritz Haber and Carl Bosch a Nobel Prize in chemistry (separately in 1918 and 1932!). The process utilises molecular nitrogen (N_2_, available in abundance (78%) in the atmosphere) and methane (CH_4_) in an economically sustainable, though environmentally expensive synthesis of ammonia (NH_3_). The ammonia produced this way is used as a raw material by the modern chemical industry for the production of most of the commonly used fertilisers, such as nitrates. The Haber-Bosch process is one of only three ways in which inert atmospheric N_2_ is converted to NH_3_, the other two being biological nitrogen fixation by prokaryotic microbes containing the nitrogenase enzyme complex, and geochemical conversion by lightening. Today crop producers world-wide rely heavily on synthetic fertilisers to enhance plant productivity; this trend seems likely to continue as a steadily rising population needs increased food mass and quality as well as renewable fuel.

The problem with the “fertiliser scenario” is that plants absorb at any one time only a small percentage of this applied supplement. The majority of it (30% to 50%) [1] is wasted and runs off into waterways causing environmental pollution on a massive scale (*i.e*., the Mississippi River Delta). In many areas algal blooms and eutrophication are huge problems. In addition the nitrogen in the soil is broken down by soil bacteria, through a process called denitrification, to N_2_O (nitrous oxide), which reacts with oxygen to give rise to NO (nitric oxide), which in turn reacts with ozone (O_3_). Natural sources of N_2_0 are soils (contributing 6.6 Tg N/year), oceans, rivers, and estuaries (contributing 5.4 Tg N/year). According to the US Environmental Protection Agency (EPA), N_2_O has about 294 times higher impact per unit mass (global warming potential) than carbon dioxide and therefore even in small quantities can contribute as GHG in a big way. Application of nitrogenous fertilisers accounts for the majority of N_2_O emissions. It has been suggested that for every 100 kg of fertiliser N added to the soil, on average 1.25 kg of N is emitted as N_2_O, which is equivalent in GHG effect to around 600 kg of CO_2_. The GHGs affect the temperature of the earth by absorbing and emitting radiation within the thermal infrared range (the “Greenhouse Effect”).

Moreover, every step in the production, delivery and application of nitrogen fertiliser requires fossil fuels. Even though formation of fossil fuels is occurring naturally through anaerobic decomposition of buried plants and animals, they are considered non-renewable as they take millions of years to form in large quantities, and reserves are being depleted much faster than new ones are being formed. The world energy consumption has increased dramatically in recent times, growing at the rate of 2.3% each year [2]. Unfortunately an estimated 86% of this energy comes from burning of fossil fuels (36% from petroleum, 27% from coal and 23% from natural gas [2]. The current demand for oil from fossil fuels is around 85 million barrels per day (about 159 litres per barrel), which is expected to rise to around 106 million barrels per day by 2030 [3]. In addition, burning of fossil fuel is considered to be the largest source of GHG emissions due to human activity, with electricity production (coal combustion), transportation (petrol, diesel and aviation fuel) and industry (gas and coal) being the major culprits.

## 2. Symbiotic Nitrogen Fixation

Plants of the Leguminosae (legume) family can provide an option for reducing our heavy reliance on nitrogen fertilisers. Legumes are unique in that they have the ability to form a symbiotic relationship with soil bacteria collectively called “Rhizobia” [1] (note that the non-legume tree Parasponia also forms nitrogen-fixing nodules with rhizobia, and actinomycete soil bacteria called ‘Frankia’ form nitrogen-fixing nodules on non-legume plants like Casuarina, Datisca and Alnus). The bacteria are housed in special root organs called “nodules” where they fix atmospheric nitrogen gas to ammonia, which the plant can assimilate via glutamine synthase to form glutamine. In return, the bacteria derive plant carbohydrates, mainly as malate for food and energy source for nitrogen fixation. The importance of this process is enormous as it reduces the plant’s and thus agriculture’s dependence on nitrogen fertilisers. It has been estimated that biological nitrogen fixation produces roughly 200 million tonnes of nitrogen annually [4,5].

It would be a huge benefit to humanity and the environment, if all agriculturally important plants could be made to fix nitrogen. This today seems unlikely as the physiological barriers are multi-fold and even despite large advances in knowledge, unknown. With this end in mind a lot of work has been carried out to understand the process of symbiotic nitrogen fixation, which can be thought of as consisting of three components; first, the formation of nodules which provide the correct environment for housing the nitrogen-fixing bacteria; second, the regulation of symbiotic tissue (*i.e.*, nodule numbers) by internal and external factors, and third, the actual conversion of atmospheric nitrogen into ammonia by the invading bacteria using the nitrogenase enzyme complex and its associated biochemical machinery.

### 2.1. Nodule Formation

**Figure 1.**
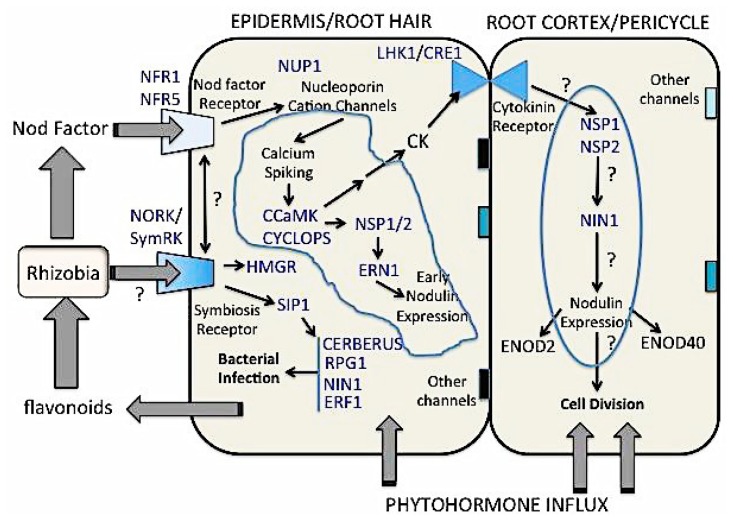
Early nodulation gene cascades in legumes. Flavonoids released by the plants are detected by compatible bacteria in the rhizosphere leading to production of Nod factors (NF), which are perceived by a dimer receptor made up of LysM type receptor kinases NFR1 and NFR5 at the root epidermis. This triggers the downstream gene cascade including those involved in nucleoporin, cation channels, calcium spiking, early nodulin expression and cytokinin signalling leading to cortical and pericycle cell divisions, and concomitant bacterial infection.

**Figure 2.**
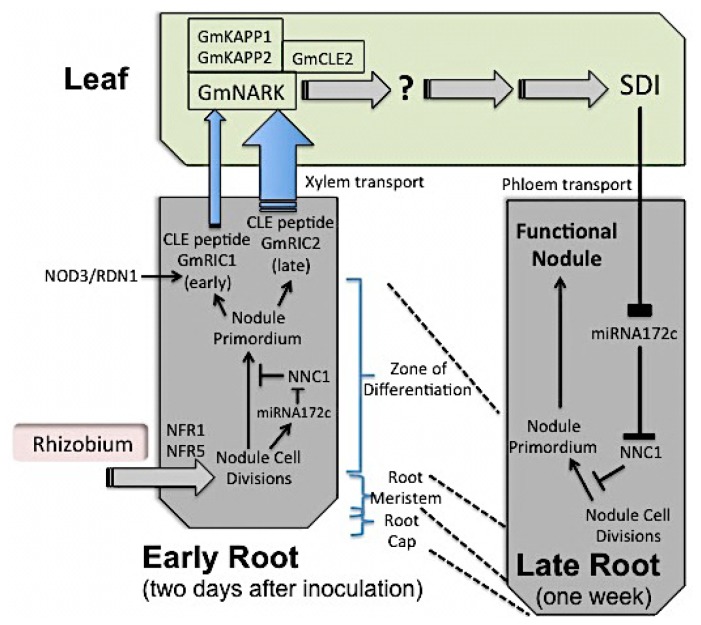
Systemic regulation of nodulation in legumes. Formation of nodule primordia (requiring NF perception as shown in Figure 1) initiates the production of miRNA172, which targets AP2 transcription factor type GmNCC1 (Nodule Number Control 1, which blocks further nodule development). Also induced are rhizobia-induced CLE peptides (such as GmRIC1 and GmRIC2 in soybean), with subsequent xylem transport to the leaves, where they act as ligands for a LRR receptor kinase (LRR-RK) like GmNARK, MtSUNN, or LjHAR1. Perception of the ligand allows for the phosphorylation of the kinase domain of the LRR receptor. KAPP1 and KAPP2 are transphosphorylated. A shoot-derived inhibitor (SDI) molecule is produced following the perception of the peptide signal by NARK, which is then transported to the roots (possibly via the phloem) where it inhibits *miRNA172*, thus leading to increased GmNNC1 and resultant nodule inhibition [33]. In *GmNARK*-deficient mutants, no degradation of the miRNA occurs, leading to continued *GmNNC1* degradation and nodule development. Note, *NOD3* and *RDN1* genes of *P. sativum* and *M. truncatula* have high similarity to peptide glycosylating genes of *Arabidopsis*, perhaps essential for arabinose addition to the 13 amino acid long CLE peptide. Mutations in *NOD3* and *RDN1* lead to super- or hypernodulation controlled by the root genotype, in contrast to mutants in *GmNARK*, *MtSUNN* or *LjHAR1*, which are shoot-controlled.

**Figure 3.**
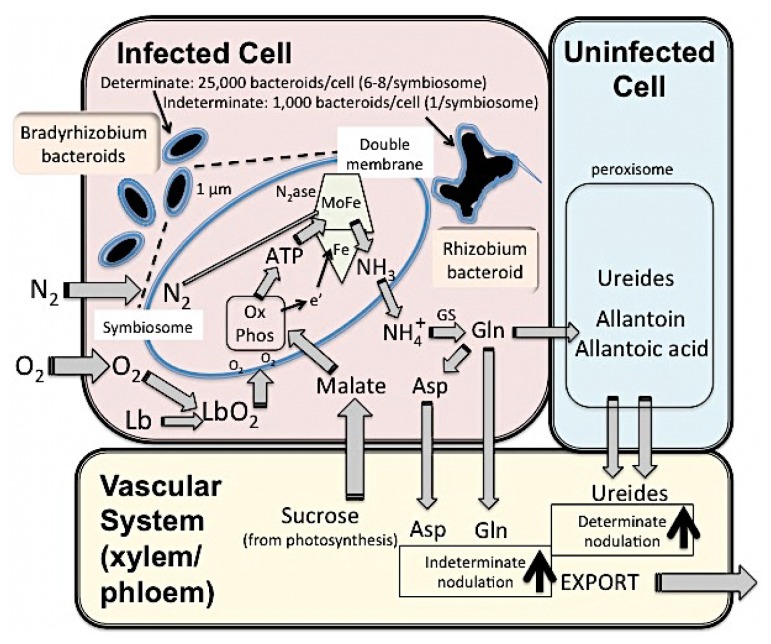
Schematic mechanisms of symbiotic nitrogen fixation in a legume nodule. N_2_ entering the symbiosome is converted to NH_4_^+^ by the nitrogenase enzyme complex of the bacteria using ATP and an electron donor. The NH_4_^+^ is then converted to glutamine via glutamine synthase (GS) and asparagine. Glutamine is exported from the infected cell to uninfected cells where in the peroxisomes ureides, such as allantoin and allantoic acid are synthesised. All the nitrogen transport molecules (Gln, Asp, ureides) are exported to and transported via the vascular system to the rest of the plant. Note that indeterminate and determinate differ in their spectrum of transport molecules. Ureides are highly beneficial as their have a 1:1 carbon:nitrogen ratio (compared to 2:1 for glutamine and asparagine).

The process of nodule formation requires a coordinated exchange of signals between the two symbiotic partners. The understanding the genetic basis of this relationship is of paramount importance [6] and essential for the optimisation of nitrogen acquisition rates in legumes themselves. Bacteria, being the “easier” experimental organism had their symbiotic genes first characterised by transposon mutagenesis; this achieved the definition of over 50 nodulation genes (*Nod* and *Nol*) in bacteria, and about the same number controlling nitrogen fixation; thus many nod^−^ and fix^−^ bacterial strains exist in many species of rhizobia. The genomes of many rhizobia, including *Mesorhizobium loti*, *Sinorhizobium meliloti* and *Bradyrhizobium japonicum* have been fully sequenced [7–9]. Work on the plant was slower because of longer life cycle and greater genetic complexity, until nodulation mutants were isolated and characterised in several legumes such as *Lotus japonicus*, *Medicago truncatula*, *Pisum sativum* (pea) and *Glycine max* (soybean) [6,10,11]. Since then, plant research on nodulation has accelerated (Figures 1–3).

Figure 1 summarises early nodulation steps as defined by plant mutations. It is known for nearly three decades that plants release signals such as flavonoids (e.g., the flavone 7,4 dihydroxyflavone and the isoflavone genistein), which are perceived as a “cocktail of variable composition” by compatible bacteria in the rhizosphere [12,13]. This activates the nodulation (*Nod*) genes of rhizobia which in turn release bacterial signals, mainly the lipochito-oligosaccharide Nodulation (or Nod) Factors (NF), which trigger early events in the nodulation process [10,14]. This molecule, existing in differently decorated versions, is similar to the chitin effectors known in plant pathogenicity. Perception of the Nod factor results in root hair curling by which the rhizobia are entrapped, initiating the formation of tubular structures called the infection thread, through which rhizobia penetrate root hair cells and adjacent cortical cells opposite the developing xylem poles [15]. Concurrently, cortical and pericycle cell divisions are induced resulting in the formation of the nodule primordium (see [16] for a detailed graphic depiction of determinate and indeterminate nodulation events). Rhizobia travel through the infection thread (by bacterial cell division) and are released into the induced nodule primordium cells [17,18]. As the nodule primordium develops into the mature nodule, bacteria are enclosed within the symbiosome membrane, derived from an inverted plant plasma membrane. Here they progressively experience lower (microaerobic) oxygen concentration and differentiate into bacteroids, fixing diffused nitrogen gas using their nitrogenase enzyme complex [15,19,20].

Nodules could either be determinate (e.g., on soybean and *L. japonicus*) or indeterminate (e.g., on *M. truncatula* and pea) depending on the site and depth of initial cortical cell division and more critically the time of active meristem termination (nodule growth). Determinate nodules are usually initiated sub-epidermally in the outer cortex (*L. japonicus* being an exception with mesodermal divisions) [21], and nodular meristematic activity is terminated early giving rise to spherical nodules whereas in indeterminate nodules cell division initially occurs anticlinally in the inner cortex, followed by periclinal divisions in the pericycle; meristems are more persistent for some time, resulting in cylindrical nodules [19,22,23].

Determinate nodules are also characterised by bacteroids that have maintained a free-living type cellular shape and size; they remain viable and can be plated. For example, about 25,000 bacteroids are found per soybean nodule cell, in contrast to about 1000, found in indeterminate nodulation species such as white clover. Interestingly, bacteroids of determinate nodulators are packaged usually between 6 to 8 per individual symbiosome, while those of indeterminate type are pleiomorphically enlarged; indeed a single bacteroid occupies a single symbiosome. These differences are explained by the continued cell division after symbiosome formation, with bacteroids of indeterminate type fail to physically separate. This failure to divide properly is caused by recently discovered ‘Nodule-specific Cysteine-rich (NCR) Peptides’.

Several genes involved in different steps of the nodulation process have been isolated and characterised (Figure 1). The perception of NF occurs through binding to two receptor-like kinases (e.g., *Lotus japonicus* and soybean Receptor kinases 1 and 5; LjNFR1 and LjNFR5), each consisting of an intracellular kinase domain, a transmembrane domain and an extracellular LysM domains [24–28]. The LysM receptor kinases are also known to interact with other proteins such as remorin proteins (MtSYMREM1) [29] and a Rho-like small GTPase (LjROP6) [30]. Several other proteins are also involved in the early NF perception, such as a transcription factor (LjSIP1), a coiled-coil protein (MtRPG) and a 3-hydroxy 3-methylglutaryl coenzyme reductase (MtHMGR1); however, their exact role is still under investigation.

Following the perception of NF several other genes such potassium ion-channels (LjCASTOR and LjPOLLUX) and nucleoporins (LjNUP133 and LjNUP85) are activated, which triggers Ca^2+^ uptake in root hairs. This calcium signal is perceived by a Calcium and calmodulin-dependent protein kinase (CCaMK, MtDMI3). The next step in the process is the activation of several transcription factors such as Nodulation Signalling Pathways 1 (MtNSP1 and MtNSP2), ERF (Ets2 repressor factor) required for nodulation (MtERN) and Nodule Inception (LjNIN), which in turn activate the expression of early nodulation (*ENOD*) genes in the epidermis.

### 2.2. Autoregulation of Nodulation

Isolation of super- and hypernodulation mutants has demonstrated that the plant controls the regulation of nodule number through a process called “autoregulation of nodulation” (AON; Figure 2) [21,31]. Such plants have abundant nodules, show nitrate tolerance in nodulation and are characterised by a large proportion of the root that has nodules (the so-called “nodulation interval”). Thus elevated nodule number per plant alone is not a good indicator of autoregulation deficit; one needs to see that increased nodulation interval. For example, the ethylene-insensitive mutant of *M. truncatula* (called “*sickle*”), mutated in the *MtEIN2* gene, has increased nodule number but still possesses AON, indicating that these two regulatory processes work in parallel [32].

It is now known that autoregulation signals (CLE peptide hormones, GmRIC1 and RIC2 (*G. max*), CLE12 and CLE13 (*M. truncatula*) and RS1 and RS2 (*L. japonicus*)) are generated in response to inoculation and early cell division in the roots of nodulating plants. This root-derived signal (called Q in the original AON model of Gresshoff and Delves [19]) is transmitted via the xylem to the shoot [34] where its perception leads to the formation of another signal, called the shoot-derived inhibitor (SDI) [19,35]. This is then transported back to the roots where it acts to inhibit further nodulation by arresting early cell division stages. Inhibition of cell division by the autoregulatory signal occurs even earlier for indeterminate nodulating legumes (like *M. truncatula*) where autoregulated regions of the root are actually devoid of visibly arrested major nodule meristems, compared to soybean. In soybean, such arrested stages are released for fast nodule formation if the existing nodules are removed.

The shoot-derived autoregulation signal (SDI) might be chemically the same in different legume species as suggested by the success of reciprocal grafts between model legumes Lotus and Medicago [36] and *Glycine max* and *Glycine soja*. One critical gene involved in the perception of the autoregulation signal encodes a Leucine-rich repeat (LRR) receptor kinase named GmNARK (nodule autoregulation receptor kinase), and is expressed most strongly in leaves of soybean and is structurally highly similar to Arabidopsis CLV1 [37–40]. Identical genes with the same phenotypic outcome in mutants were discovered in *L. japonicus* (*LjHAR1;* 21,37,39), *M. truncatula* (*MtSUNN*) and pea (*PsSYM29*). Other genes such as *LjCLV2*, *LjKLAVIER* and those encoding kinase associated protein kinase (GmKAPP1 and GmKAPP2) and a putative Ubiquitin Fusion Degradation Protein (GmUFD1a) are involved in the AON pathway [41]. All of this indicates long and short distance communication among different parts of the plant [6,11,42].

*GmNARK* is also involved in the inhibition of nodulation by nitrate, as deficient mutants show the ability to nodulate under inhibitory conditions. Nitrate also induces a specific CLE peptide (GmNIC1 in soybean, or non-specifically in conjunction with rhizobial inoculation via LjRS1 in *Lotus*) to regulate nodulation progress. The nitrate-inhibition process, however, is root-specific and links the classical data showing: (a) *GmNARK* mRNA expression being high in roots and shoots [43]; and (b) localised not systemic control of nodulation in legume split roots.

### 2.3. Nitrogen Fixation

The actual process of conversion of atmospheric nitrogen (N_2_) to ammonia (NH_3_) takes place inside the nodule symbiosome by the bacteroids using the nitrogenase enzyme complex, which is extremely oxygen-sensitive and is thus irreversibly damaged by oxygen (Figure 3). The microaerobic environment inside the symbiosome is provided by nodule tissue buffering, coupled with high respiratory bacteroid activity. Additionally oxygen binds to the monomeric leghemoglobin protein (highly related to mammalian myoglobin containing a heme group carrying iron), which, just like its highly related animal hemoglobin molecule, functions as an oxygen gas transporter rather than a protective screen.

The nitrogenase enzyme is composed of two metalloproteins. The first (a dinitrogenase molybdenum-iron (MoFe) protein) is a 220,000 Da tetramer composed of two non-identical subunits (α and β), and the second (a dinitrogenase reductase Fe protein) is a 68,000 Da dimer formed of identical subunits. The α subunits of the MoFe protein bind two iron-molybdenum co-factors (FeMoCo). In addition, two other groups containing 4Fe–4S clusters bridge the α and β subunits by covalently binding to the cysteine residues of the MoFe protein. A third type of Fe–S group is also linked to the Fe protein [44–46]. The nitrogenase enzyme complex requires adenosine triphosphate (ATP) and an electron donor. The electrons are transferred to the Fe protein of nitrogenase by a 4Fe–4S, beginning a series of oxido-reduction cycles. The reduced Fe protein binds to two molecules of MgATP, which are hydrolysed to drive an electron from the Fe protein to the MoFe protein. It is on the MoFe protein that the actual reduction of N_2_ gas occurs.

Electron transfer occurs six times for each fixed N_2_ molecule so that 12 ATPs are required to fix one N_2_ molecule (see Equation (1)). In addition, nitrogenase also reduces protons to H_2_ gas consuming two electrons. Therefore, each N_2_ reduction requires eight electrons to be transferred and 16 MgATPs hydrolysed [47]. The universal reaction is:

(1) $${\text{N}}_{2}+8{\text{H}}^{+}+8{\text{e}}^{-}+16\text{ATP}↔2{\text{NH}}_{3}+{\text{H}}_{2}+16\text{ADP}$$

Ammonia, usually as ammonium in the aqueous cytoplasm, and glutamate are combined by glutamine synthase to form the universal nitrogen source glutamine. This in turn is either converted to asparagine or exported to the adjoining uninfected cell of determinate nodulating legumes like soybean to be converted in its peroxisome to the ureides, allantoin and allantoic acid (see Figure 3). All four nitrogenous compounds serve as nitrogen source for the remainder of the plant; different legume types and environmental conditions may vary the xylem content of these compounds.

## 3. *Pongamia pinnata*, a Legume Tree with Biofuel Potential

### 3.1. Renewable Energy

There are multiple forms of renewable energy, as they come in the form of electricity, heat and fuel. Electrical energy is generated most commonly generated by burning of fossil fuel (either coal, oil or natural gas), hydroelectric power generation, or heat from nuclear fission. But these mechanisms are not renewable (oil and gas will run out, dams will fill by sedimentation, nuclear fission relies on uranium) and thus lack prolonged sustainability. Renewable energy can also be captured by photovoltaic cells harvesting solar radiant energy, or wind turbines (harvesting atmospheric wind movement). These sources are excellent but are limited by lack of constancy and prediction of supply. This is coupled with the technical difficulty of storing large amounts of electrical energy. As such these renewable energy sources are currently valuable and of interest, but for the long term need to be combined with more consistent alternatives.

Renewable fuels (*i.e*., storable chemical energy) are such a component. There are again numerous sources, ranging from plant biomass-to-fuel conversion, algal biodiesel, waste conversion to gas, and crop plant biofuel. An essential aspect of any such fuels is the overall energy balance and industrial efficiency in terms of capability and scale. Related to the energy balance is a complete Life-Cycle-Analysis (LCA), literally looking at the processes and related industry from “cradle-to-grave”.

Many current plant biofuel feedstocks such as oil palm, canola, willow, corn (*Zea mays*), sugarcane, jatropha, sorghum and even algae may produce abundant fuels but are not nitrogen-fixing [48]. Some sugarcane varieties have been shown to display some biological nitrogen fixing activity through association with endophytic diazotrophic bacteria like *Azospirillum* and *Azotobacter* [48,49]. Thus nitrogenous fertiliser is the major energy input, as the Haber Bosch process is heavily based on fossil fuel for the required methane, heat and pressure [50].

Thus, to achieve a degree of sustainability for renewable energy production the feedstock needs to absorb a literally inexhaustible supply of energy (say solar radiation via plant photosynthesis), coupled with a renewable source of nitrogen fertiliser via the natural processes of symbiotic nodulation and nitrogen fixation. Even phosphorus can become limiting and may require an effective symbiosis with soil fungi broadly called “mycorrhizae”. Only then can the process be called “renewable”. As an example, Chandrashekar *et al.* [51] published a preliminary LCA of Pongamia grown in a defined area of India. The results demonstrated clearly that the leguminous nature gave the plant a major advantage over the highly popularised non-legume jatropha tree.

### 3.2. Pongamia Biology

**Figure 4.**
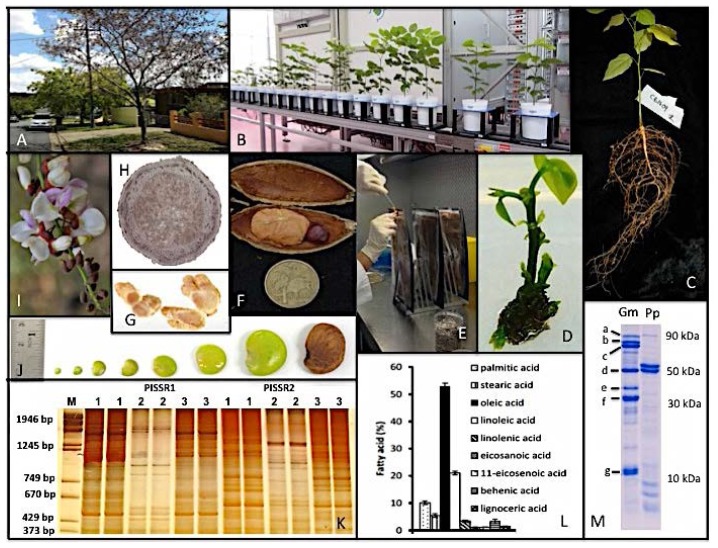
The biological properties of Pongamia, a biofuel feed stock tree with potential. (**A**) Street trees in Brisbane (Australia) during spring (late November). Note the variable spring leaf burst caused by genetic heterogeneity. Such variation is a problem for the producer, but a benefit to the experimenter; (**B**) Pongamia plants grown for analysis in the Adelaide Automated Growth Facility; (**C**) Pongamia seedling plant with nodules induced by *Bradyrhizobium japonicum* strain CB1809, a common Australian soybean inoculum; (**D**) Pongamia growing in tissue culture. Clonal regenerants from immature cotyledons are now growing in fields; (**E**) Pouch growth of Pongamia seedlings allows nodulation studies; (**F**) Pongamia pod and seed (about 20 mm long seed; about 2 g/seed). Pod casing potential can be co-fired for electricity formation; (**G**) Pongamia nitrogen-fixing root nodules (about 2–3 mm in length four weeks after inoculation); (**H**) Pongamia root nodule close-up (about 3 mm in diameter). Bacteria are housed as bacteroids in the interior; (**I**) Pongamia flowers (pea-like; about 6–8 mm in length); (**J**) Pongamia seed development: Takes 10–12 months from fertilisation to harvest of dry seed; (**K**) Pongamia genetic diversity testing. PISSR markers (Pongamia Interstitial Single Sequence Repeats) based genomic DNA sequence separated by polyacrylamide gels electrophoresis and silver stained (Bassam and Gresshoff, Nature Protocols, 2007); (**L**) Pongamia seed oil composition derived from one seed of tree HS-62; (**M**) Pongamia seed cake components stained for protein. Left lane: soybean (Gm); right: Pongamia (Pp). (a) lipoxygenase (90 kDa); (b) 7Sα prime (70 kDa); (c) 7Sα (66.4 kDa); (d) 7Sβ conglycinin (51 kDa); (e) 11SAβ (41 kDa); (f) 11SA1a, A1b, A2 (38.5 kDa); (g) sB1a, B1, B2, B3, 11SB4 (10 kDa). The genes for the main *Pongamia pinnata* seed storage proteins (50 and 52 kDa) were cloned and shown to be similar to the low quality seed storage protein 7S β conglycinin.

Pongamia stands out amongst all the currently popular biofuel feedstock plants, as being a high seed yielding, nitrogen-fixing legume puts it in a more sustainable position (Figure 4). A medium-sized, fast growing tree-legume, native to Southern and South-east Asia including northern Australia, *Pongamia pinnata* (also known as *Millettia pinnata*) has been gaining popularity in recent times due to its oil-rich seeds (40% to 50%). The oil is non-edible and resultant seed-cake finds utility as supplemental animal feed only in low portions (10%–20%). First flowering starts as early as 16 months after sowing [45] but reaches an economically consistent scale only after 5 years. Pongamia trees are capable of producing 15,000 to 20,000 seeds per year and with an average mass of 1.5 g per seed; this equates to between 22 and 30 kg of seeds per tree per year. Seasonal variation occurs. Larger seeds (2.8–3.0 g dry weight per seed) and seed crops (estimated at 30–50,000 seed per mature tree) have been recorded in the South Eastern Queensland area [48].

The seeds contain triglycerides high in the mono-unsaturated fatty acid, oleic acid (C18:1; 45% to 55%) and low in saturated fatty acids such as palmitic acid (C16:0) and stearic acid (C18:0), a composition ideal for biodiesel production. This commercially valuable fuel is obtained by the simple process of transesterification with CH_3_OH (methanol) in the presence of KOH (potassium hydroxide) catalyst to meet current industry standards, European EN14214 and U.S. ASTM D 6751-08 [52]. At a conservative estimate of 40% (*v*/*v*) oil per seed from a 15,000 seed per tree harvest equates to 9 and 12 L of oil per tree per year, and with a planting density of 400 trees per hectare this would yield between 3600 and 4800 L of oil ha^−1^·year^−1^. Recent silvicultural advances coupled with mechanistic harvesting technology suggest a possible optimal planting rate at 600–800 trees per hectare. Currently research is underway looking at aspects of tree spacing on harvest yield and seed quality.

Pongamia grows best in humid tropical and sub-tropical climates; however, it can tolerate a wide range of temperatures (minus 5 to 16 °C minimum and 27 to 50 °C maximum) and rainfall (500 to 2500 mm) [53–55]. It has been reported to be drought- and salinity-tolerant [56,57] and hence can be grown on marginal land unsuitable for food crops. Slightly salinated water can be used on occasions for irrigation, especially at the seedling stages, when the root system has not penetrated deeper soil layer, richer in a constant water supply. Extremely high summer temperatures (>40 °C) for prolonged periods (days) appear to be negative for seed development.

These are valuable traits of Pongamia in today’s world where starvation and malnourishment are real problems and destined to get worse with the rising population, that it can be grown without competing for land, water and labour with food crops. Its genetic outcrossing nature gives rise to a wide range of phenotypes in tree architecture, leaf size and shape, seed size and shape as well as oil quality and quantity [58]. This ensures a large pool of germplasm to select from but also poses the challenges of clonal propagation of selected elite material. Here Pongamia will benefit from “borrowing” knowledge from other tree species of interest such as fruit and nut trees.

Genetically little is known of *Pongamia pinnata*. It is a true diploid with 2*n* = 22. The genome size [59] is estimated at 1300 megabases/haploid genome. The tree produces extensive flower formation in spring to early summer. Insects such as bees fertilise flowers leading to the formation of pods that have two ovaries, but only occasionally two smaller sized seeds. The species appears to be outbreeding, causing extensive genetic variability in off-spring [58] (see Figure 4). However, seedlings derived from a common mother tree are more similar to each other than unrelated trees.

Clonal propagation by vegetative rooted cuttings is feasible and has laid the basis of an emerging Pongamia biofuel industry. Preliminary tests have demonstrated the ability of tissue culture (*in vitro*) propagation; immature cotyledon-derived clones have been produced efficiently (see Biswas *et al.* [48] for illustrative figure) and are being testing in three different geoclimatic environments in Queensland; this will ascertain aspects of genotype x environment interaction.

*Pongamia pinnata* has already served as a source for numerous biodiversity studies. Jiang *et al*. [58] developed ISSR molecular markers to evaluate DNA sequence diversity. Simple shoot and root transcriptome databases are available [60] as is an Illumina^®^ genomic database in the authors’ laboratory; this is hopefully the starting platform for eventual complete genome assembly and analysis. Already the organelle genomes of the mitochondrion and the chloroplast have been determined, providing an experimental entry point into the cellular structures responsible for critical photosynthesis and respiration [61]. Significantly the chloroplast genome (152,968 bp) is gene-rich as in other legumes; even the gene order, orientation and number are conserved. The larger mitochondrial genome (425,718 bp), in contrast, appears to more gene-poor and is variable in size compared to that of *Vigna radiata* and the model legume *Lotus japonicus* (size 380,861 bp).

### 3.3. Nodulation and Nitrogen Fixation in Pongamia

Even though little research has been carried out on Pongamia nodulation, everything points to the fact that general properties of the Pongamia process resemble those of better-studied annual crop legumes such as soybean and bean (*Phaseolus vulgaris*). However, unlike a lot of other legumes, which form symbiotic relationships with one or a select few rhizobia, Pongamia can nodulate with several strains of both *Bradyrhizobium* and *Rhizobium* [62–64]. However, in a bid to select superior strains as inocula, which could promote growth and potentially increase yield of the oil-rich seeds, several strains were tested. *B. japonicum* strains CB1809 and USDA110 (common soybean inocula) were identified as the most effective [62]. Nodules formed on seed-derived seedlings within about 4 weeks with clear nodulation and established symbioses by 8 weeks at 28 °C. The nodules produced by these strains were larger, and more uniformly filled with infected bacteroid zones. In contrast, the nodules produced by the less effective strains had several lobed infection zones with variable bacterial occupancy [65]. The internal structure of the nodules resembles a typical legume nodule with a central infected zone surrounded by vascularised cortex. The Pongamia nodules actively fix nitrogen as demonstrated by the quantification by gas chromatography of ethylene in the acetylene reduction assay, where C_2_H_2_ (acetylene) served as a substrate for bacterially encoded nitrogenase [65]. However, it may be valuable to develop other methods of nitrogen fixing capability, such as isotope dilution methods, as acetylene reduction is weakened by experimental errors such as the variable oxygen barrier.

Pongamia nodules have been reported to be determinate in nature with a spherical morphology [53,66,67]. However, it was later shown that they start of as such spherical determinate-like structures (Figure 4H), but as the plant initiates new cell divisions, turns determinate-type nodules into more meristematic-type structures [65]. A mature Pongamia tree therefore displays a combination of spherical and larger coralloid structures (Figure 4G), consistent with observations in other tree legumes [68,69].

*Pongamia* plants also displays autoregulation of nodulation (AON) and nitrate (NO_3_) inhibition of nodulation, demonstrated by split root experiments on *Pongamia* seedlings [65]. Inoculation with rhizobia of one portion of the roots system systemically suppresses nodule formation on a later inoculated root portion. Likewise seedlings inoculated at planting are characterised by significant nodule formation on the upper portions of the root system, while lower parts are nodule-free (Figure 4C). This is identical to the situation of annual legumes such as soybean. One concludes that despite being a multi-season legume tree, the *Pongamia* root symbiosis is very similar to that seen in annual legumes. Many questions still remain, demonstrating variation induced by biodiversity.

Proponents of nitrogen fertilisers have debated that biological nitrogen fixation also results in N_2_O emissions through soil rhizosphere as well as nodules. Their arguments are usually based on studies undertaken in the 1980s where relatively high N_2_O emissions were recorded for commonly grown legumes such as alfalfa and soybean [70,71]. However, later studies showed that the source of these emissions was carry-over nitrogen fertiliser, and when these background levels were omitted, the emissions from legume fields were lower than fertilised grasslands and non-legume fields. In an experiment carried out over two years it was shown that even the highest emissions from soybean were substantially lower than that from maize grown with high rates of nitrogen fertiliser [72].

## 4. Conclusions

Today growth of food and biofuel plants relies heavily on nitrogen fertiliser, production of which is dependent on fossil fuels. However, the real danger of depletion of existing fossil fuels, associated cost increases in the depletion period, and the environmental impact of their use is making it important to look for alternatives to synthetic fertilisers. Symbiotic nitrogen fixation by legume plants provides such an alternative. Legumes are unique in that they have the ability to form a symbiotic relationship with nitrogen-fixing bacteria (collectively called rhizobia), which are housed in special root organs called nodules (NB, there are other nitrogen-fixing symbioses with plants and the bacterium called “Frankia”, *i.e*., with the non-legume casuarina or elm trees. This field of investigation has been highly descriptive, since the Frankia bacterium is difficult to handle microbiologically).

Several strategies to transfer the process via inter-specific gene transfer to non-legumes like rice and corn are being pursued (reviewed by [73]). The fact that the more common mycorrhizal fungus symbiosis shares many common signaling elements (SymRK to CCaMK) with the rhizobial symbiosis gives hope that transfer of the additional elements through biotechnological methods may be possible. However, the transfer to a monocot non-legume may stretch the possibilities as the inherent phytohormone regulation of monocot and dicot is highly divergent (*c.f.*, the differential auxin herbicide toxicity spectrum). Perhaps it would be easier to consider nodulation of non-legumes like potato or canola, (a) because they are dicot and (b) because other dicot non-legumes nodulate and fix nitrogen (e.g., *Parasponia* and the *Frankia*–nodulated plants). The most important target for genetic engineering is the Nod-factor receptor (NFR1), since Nod-factors as well as the Myc-factors, both being lipochito-oligosaccharides suggest a broader recognition spectrum. Several nodulation-associated transcription factors are also being targeted.

Another more ambitious approach is to introduce the nitrogenase enzyme complex into plant cells via chloroplast transformation. The resulting “nitroplast” would harvest solar energy, but if inactivated by mutation for Photosystem II would not yield abundant oxygen. This concept was already developed by John Postgate, Ray Dixon and associates nearly 40 years ago; however, despite elegant biotechnological approaches, no significant advances have been achieved. Success in any of these methods will have a drastic impact on the way food and biofuel plants are grown.

However, these methods are still some way away, and in the meantime we propose the use of non-food legumes for direct biofuel production and companion cropping. In addition to being a legume, it is important for a biofuel feedstock not to compete with food crops for land, water and labour. *Pongamia pinnata* is such a plant, as being drought- and salinity-tolerant; it can grow on marginal land not suitable for most food crops. Globally such land areas are abundant. However, more importantly, being a legume means that it does not require supplied nitrogen fertilisers, thereby increasing its sustainability.
